# Elective Robotic Splenectomy for Wandering Spleen‐Associated Pancreatitis: A Novel Case Report

**DOI:** 10.1002/ccr3.72385

**Published:** 2026-04-03

**Authors:** Mohamed Nasser Elshabrawi, Ismail Elkhattib, Dina Elraggal, Bassant Elsayed, Neil Hansen, Faruq Pradhan

**Affiliations:** ^1^ Department of Clinical Research Aswan Heart Center, Magdi Yacoup Foundation Aswan Egypt; ^2^ Division of Gastroenterology and Hepatology University of Nebraska Medical Center Omaha Nebraska USA; ^3^ Faculty of Medicine University of Alexandria Alexandria Egypt; ^4^ Faculty of Medicine Ain Shams University Cairo Egypt; ^5^ Department of Radiology University of Nebraska Medical Center Omaha Nebraska USA

**Keywords:** case report, pancreatitis, robotic splenectomy, splenic torsion, wandering spleen

## Abstract

Wandering spleen (WS) is a rare congenital anomaly characterized by abnormal mobility of the spleen due to laxity of the splenic suspensory ligaments. WS can result in complications such as torsion, infarction, and involvement of adjacent structures including the pancreas. This report details a unique case of WS complicated by acute pancreatitis due to splenic torsion. A 45‐year‐old multiparous woman with a history of psoriasis, gastro‐esophageal reflux disease, and chronic pain presented with recurrent left upper quadrant abdominal pain and biochemical signs of pancreatitis. Cross‐sectional imaging revealed splenomegaly, abnormal spleen positioning, and torsion of the splenic pedicle with a transient elevation in lipase. A multidisciplinary team diagnosed intermittent splenic torsion and detorsion complicated by traction on the pancreatic tail, leading to pancreatitis. Despite the recurrent nature of her symptoms, the patient remained clinically stable, allowing for elective robotic splenectomy. Intraoperatively, the spleen was found in the pelvis. Notably, the tail of the pancreas was also mobile and displaced from its normal location, necessitating a pancreatopexy to anchor and secure the body and tail of the pancreas within the left upper quadrant, thereby restoring normal anatomy and reducing the risk of future displacement. Wandering spleen can be a rare but important cause of recurrent abdominal pain and acute pancreatitis. Timely recognition of its imaging features and complications, such as splenic torsion and pancreatic inflammation, is essential. Elective splenectomy or splenopexy should be considered for patients with preserved splenic function and stable clinical status.

## Key Clinical Message

Wandering spleen should be considered in patients with recurrent abdominal pain or pancreatitis with abnormal splenic position. Early recognition of characteristic imaging findings permits elective operative management, prevents complications, and avoids emergency splenectomy.

## 1 Introduction

Wandering spleen (WS) is a rare developmental anomaly, with a global incidence of less than 0.2% [1]. It results from congenital underdevelopment or acquired laxity of splenic suspensory ligaments which normally anchor the spleen in the left upper quadrant [2]. This aberrant mobility is associated with a long, tortuous splenic vascular pedicle, increasing the risk of splenic torsion and infarction [3].

WS exhibits a bimodal age distribution affecting children below the age of 10 years and women of reproductive age, particularly multiparous females. Clinical presentations vary widely, from incidental findings on imaging to an abdominal or pelvic mass, chronic recurrent or acute abdominal pain, or less commonly present with complications involving adjacent structures [2, 4]. One such complication is acute pancreatitis due to simultaneous torsion of the pancreatic tail, which lies in close proximity to the splenic hilum [4, 5].

Management of WS depends on patient presentation and splenic viability. Uncomplicated cases are managed with splenopexy, in which the spleen or its hilum is fixed directly to the abdominal wall or diaphragm. However, if splenic viability is irreversibly compromised or complications are present, splenectomy is indicated [6]. Therefore, early diagnosis of WS is essential to preserve organ function and avoid life‐threatening complications [6, 7].

Herein, we report a rare case of WS in a 45‐year‐old woman with intermittent abdominal pain complicated by acute pancreatitis. She is planned to undergo an elective robotic splenectomy.

This case was reported in agreement with the principles of CARE guidelines for case reports [8].

## 2 Case History/Examination

A 45‐year‐old multiparous woman with a history of psoriasis (managed with topical corticosteroids), gastroesophageal reflux disease (on proton pump inhibitors and sucralfate), and tramadol use for chronic back pain, presented with severe acute‐on‐chronic left upper quadrant (LUQ) abdominal pain radiating to the back. She reported similar, though milder, intermittent episodes dating back to adolescence. She had previously been informed of a “hypermobile spleen” which was noted to be in a lower than usual position on imaging. No surgical intervention was undertaken, and the finding was managed conservatively with clinical observation. Over subsequent years, the patient continued to experience intermittent abdominal pain prompting one additional abdominal imaging few years before which confirmed persistent abnormal splenic position. No surgical or procedural interventions were performed until her recent evaluation and management.

## 3 Differential Diagnosis, Investigations and Treatment

**FIGURE 1:**
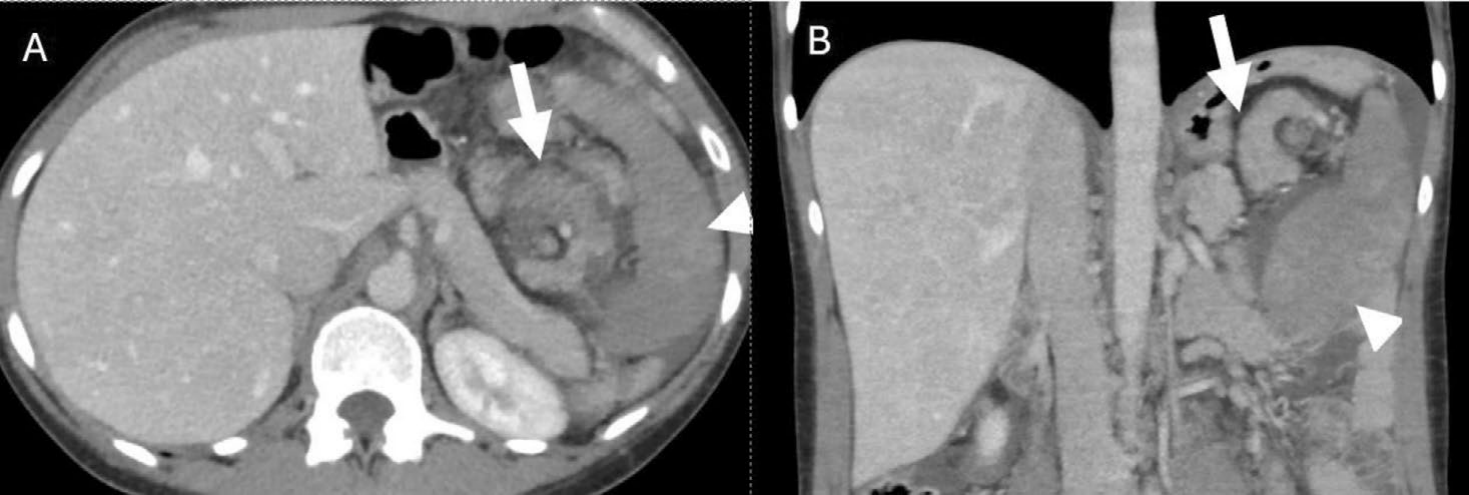
(A) Axial and (B) coronal contrast enhanced CT images showing torsion of the pancreatic tail (arrow) with swirling around the splenic hilum with reduced enhancement consistent with ischemia. The spleen (arrowhead) is enlarged, inferiorly rotated and heterogeneously enhancing due to compromised perfusion.

On presentation to the emergency room, she was hemodynamically stable. Laboratory investigations revealed an elevated lipase of 900 U/L (reference range: 0–160 U/L). Liver enzymes and complete blood count were within normal limits. A Computed Tomography (CT) scan of the abdomen and pelvis demonstrated splenomegaly and an abnormally positioned spleen with signs of acute pancreatitis (Figure 1). She was treated conservatively and discharged home.

**FIGURE 2:**
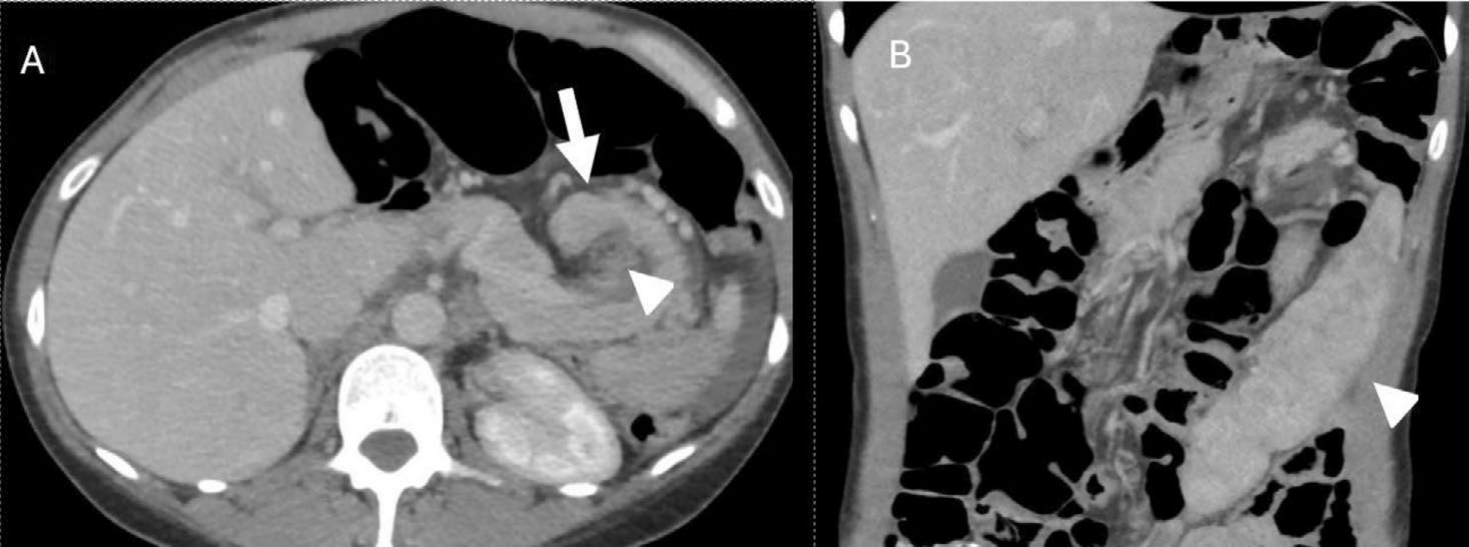
(A) Axial and (B) coronal contrast enhanced CT images. (A) Partial detorsion of pancreatic tail (arrow) with reduced swirling at the splenic hilum and improved enhancement. Fatty infiltration related to pancreatitis is improved (arrowhead). (B) The spleen (arrowhead) remains enlarged but shows partial detorsion and decreased swirling, pelvic position and improved enhancement.

Given ongoing pain symptoms after discharge, a repeat CT scan of the abdomen and pelvis (Figure 2) 2 days later showed persistent but reduced torsion at the splenic hilum. The spleen remained enlarged but also has partial detorsion occurred, with a more pelvic location and improved enhancement. A CT angiogram scan of the abdomen and pelvis performed that day revealed a markedly enlarged spleen (17.7 cm) with aberrant positioning, rotation of the splenic hilum, and torsion of the splenic artery. Despite this, splenic and portal venous flow was preserved. The pancreas showed no ductal dilation or peripancreatic inflammation, though the tail appeared adjacent to the area of vascular torsion.

An esophagogastroduodenoscopy conducted that same week showed a grossly normal mucosa throughout, with mildly dilated and partially rotated gastric body and antrum. Biopsies were negative for *H. pylori* or acute pathology.

Given her history and imaging findings, a multidisciplinary surgical evaluation concluded that her symptoms were consistent with a wandering spleen undergoing intermittent torsion and detorsion. The association with transient lipase elevation was attributed to traction on the pancreatic tail, given the close anatomical relationship.

**FIGURE 3:**
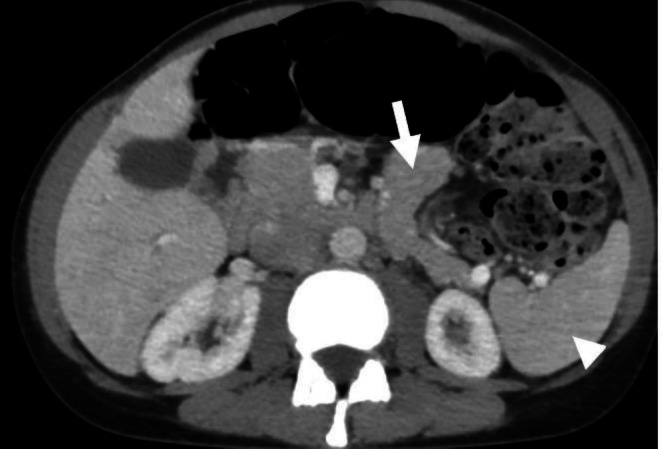
Axial contrast enhanced CT scan. The tail of the pancreas (arrowhead) has returned to a normal anatomic position with normal enhancement and no residual torsion. Similarly, the spleen (arrowhead) has returned to the left upper quadrant without ischemic change, though it remains enlarged.

A follow‐up CT scan of the abdomen and pelvis 2 months later demonstrated that both the spleen and tail of the pancreas have returned to normal positions (Figure 3).

Review of prior imaging from a few years earlier revealed a pelvic spleen measuring 17.3 cm with an intact vascular pedicle and a 15 mm cyst, consistent with longstanding ectopia.

## 4 Conclusion and Results (Outcome and Follow‐Up)

Given the presence of unexplained splenomegaly (spleen was 18.3 cm at the time of decision), thrombocytopenia (platelet count was 134 × 10^9^/L) in conjunction with spontaneous splenic torsion and detorsion, consensus among multiple consulting surgeons was that splenectomy represented the most appropriate management option in this case.

The patient remained clinically stable, allowing for elective robotic splenectomy. Intraoperatively, following a failed attempt to visualize the spleen in the left upper quadrant, a systematic laparoscopic survey was performed to locate the organ. To locate the spleen, the splenic flexure was retracted toward the caudal aspect of the body to expose Gerota's fascia and the renal capsule. Ultimately, the spleen was found to be located within the pelvis, tethered by stretched peritoneum covering the pancreatic tail. Controlled mobilization of the spleen and pancreas back to the left upper quadrant was facilitated with gentle tension applied with the use of Hunter graspers, allowing for docking the robot and for definitive splenic dissection. The pancreatic tail was mobile and required pancreatopexy after detorsion.

The patient was discharged on postoperative day one in stable condition. She experienced mild incisional soreness and fatigue during the first several postoperative days, both of which resolved without specific intervention. She resumed normal daily activities while avoiding strenuous exertion and returned to work by postoperative Day 5. At her 1‐month follow‐up visit, she remained asymptomatic with no reported complications.

## 5 Discussion

This case underlines the importance of considering rare mechanical and congenital causes for recurrent abdominal pain and pancreatitis, especially when imaging shows visceral malposition.

We highlight a rare but clinically significant variant of WS, which presents with recurrent pancreatitis due to chronic intermittent torsion and detorsion. Unlike most previously reported emergency cases, our patient maintained splenic perfusion, allowing for an elective robotic splenectomy. While torsion of the splenic pedicle can result in splenic infarction, hemorrhage, or abdominal mass, involvement of adjacent structures, especially the pancreas, is exceedingly rare [7]. Our case is particularly unique highlighting the potential for elective surgical management of WS‐induced pancreatitis in contrast to prior studies that describe WS as a surgical emergency.

Of note, in our case, intraoperative localization of the spleen was challenging, as it was found deep within the pelvic cavity rather than its anticipated position. This required additional operative time and, importantly, the extreme mobility of both the spleen and pancreatic tail necessitated a pancreatopexy to anchor and secure the pancreas in the left upper quadrant, underscoring the technical challenges that can arise even in an elective robotic setting.

The spleen is normally supported in the LUQ by the gastrosplenic, splenorenal, and splenocolic ligaments. Congenital or acquired laxity of these peritoneal attachments of the spleen results in splenic hypermobility, leading to a wandering or ectopic spleen. The most common cause appears to be a failure of fusion of the dorsal mesogastrium during the fifth and sixth week of development, resulting in an unusually long splenic pedicle. Wandering spleen has also been seen in disorders where there is failure of foregut rotation and fusion of the dorsal mesogastrium, such as prune‐belly syndrome [9]. The increased association of wandering spleen during pregnancy and in the multiparous female has led some to suggest that hormonal influences affect the laxity of the splenic ligaments [10, 11]. The long pedicle predisposes to torsion, reported from 90° to over 2000° [12]. Symptoms may be limited or absent for a long time, but complications of abdominal pain may arise as the spleen rotates around its vascular peduncle [13, 14].

Our patient presented with recurrent LUQ pain and pancreatitis without hepatobiliary or metabolic etiology, representing a milder form compared to previous cases complicated by infarction or hepatic vascular compromise [10]. The intermittent symptoms in our patient are best explained by partial torsion with spontaneous detorsion, which allows splenic reperfusion but intermittently compromises the pancreatic tail, explaining the transient pancreatitis [15, 16].

A 20‐case report review of WS (Table 1), showed the youngest patient was 3 years old while the oldest was 98, of which, 17 cases presented with pancreatic tail involvement. The spleen was infarcted in 10/20 (50%) of the cases. Nineteen patients underwent surgery with 11 (57.9%) described as emergency surgeries. Torsion degree and symptom duration are the main prognostic determinants. Partial torsion (< 360°) was usually associated with mild, reversible pancreatitis and splenic viability, whereas severe torsion (720°–1080°) almost invariably caused infarction requiring splenectomy [4, 16, 26, 28]. Pancreatic tail involvement was common, but pancreatitis was generally mild and self‐limiting, with rapid biochemical and symptomatic resolution once torsion was corrected [7, 11, 14]. Imaging, particularly the “whirl sign,” was diagnostic in >90% of cases, and associated organ involvement (e.g., gastric volvulus, colonic obstruction) often dictated surgical urgency [15]. Predisposing factors such as multiparity, pregnancy, Fragile X, and Prader–Willi syndromes further support the role of congenital or acquired ligamentous laxity. Overall, outcomes depend on splenic viability and torsion degree, while pancreatitis is secondary, transient, and reliably resolves after surgical correction [17, 18, 19, 20, 21, 22, 23, 24, 25, 27, 30]. Since 2023, we identified five cases of wandering spleen documented in the literature, four of which required emergency open splenectomy due to splenic torsion and infarction [25, 26, 27, 28, 29].

**TABLE 1:** Review of prior WS case reports including their presentation, radiologic findings and management.

Year	Study (Year)	Age/Sex	Presentation	Pancreatic tail involvement	Whirl sign	Degree of rotation	Spleen viability	Surgical timing	Surgical approach	Outcome	Comorbidities & associated factors
1984	Sheflin et al. 1984 [30]	33 F	Acute abdomen	Yes (distal pancreas)	Yes (CT swirl)	—	Infarcted	Emergency	Open, splenectomy ± distal pancreatectomy	Recovered	—
1994	Murray 1994 [17]	3.5 M	Abdominal pain	—	—	360° twist	Preserved	Elective	Splenopexy	Recovered	Fragile X $
1995	Menendez et al. 1995 [18]	98F	Acute pancreatitis	Yes	Yes	—	Preserved	Conservative	—	Improved on conservative ttt	
2003	Gilman et al. 2003 [19]	24 Pregnant	Pancreatitis. Incidental thrombocytopenia	Yes	—	180° on its pedicle	—	Elective	Splenectomy	Recovered	Pregnancy
2008	Lebron et al. 2008 [11]	20 F	Recurrent pancreatitis	Yes	Yes	—	Preserved	Elective	Laparoscopic splenectomy	Recovered	
2011	Magno et al. 2011 [20]	3 M	Vomiting, abdominal swelling, and acute pancreatitis		Yes	—	Preserved	Elective	Laparoscopic Splenopexy	Recovered	Gastric outlet obstruction
2012	Choudhary et al. 2012 [15]	87 F	Abd pain + obstruction	Yes (colon + tail)	Yes	—	Infarcted	Emergency	Splenectomy + colectomy	Recovered	Colon obstruction& constipation for years
2014	Gorsi et al. 2014 [21]	16 M	Acute pancreatitis	Yes	Yes	—	Variable	Emergency	Surgery per volvulus	Recovered	Gastric volvulus
2014	Sharma et al. 2014 [4]	27F	Acute abdomen	Yes (tail involvement)	Yes	360° twisting of the splenic hilum around the tail of her pancreas	Infarcted	Emergency	Open splenectomy	Recovered	Gastroesophageal reflux disease
2016	Loh et al. 2016 [16]	22 F	Pancreatitis + DKA	Yes	Yes	720 degrees torsion of the long splenic pedicle.	Preserved initially	Elective then emergency	Splenectomy	Recovered	Prader‐Willi $ type 2 diabetes mellitus
2018	Lourdusamy et al. 2018 [22]	28 F	Mild abdominal pain associated with nausea for two days		Yes	—	Preserved	Elective	Splenopexy	Recovered	Multiparous
2019	Colombo et al. 2019 [7]	18 F	Recurrent LUQ pain	Yes	Yes	—	Mixed infarct	Elective	Laparoscopic splenectomy	Recovered	
2021	Molinelli et al. 2021 [23]	24 F	LUQ pain	Yes	Yes	360° counterclockwise	Variable	Emergency	(Laparoscopic or conventional)	Recovered	
2021	Saldívar‐Martínez et al. 2021 [24]	43F	Pancreatitis + infarct	Yes	Yes	Double rotation (two complete twists) of the splenic hilum and gastro‐splenic ligament	Infarcted	Emergency	splenectomy + distal pancreatectomy + lumbar hernia repair	Recovered	Post traumatic Grynfelt‐Lesshaft hernia
2022	Safaya et al. 2022 [14]	25 M	Pancreatitis	Yes	Yes	—	Preserved	Elective	Splenopexy	Asymptomatic	
2023	Lakmal et al. 2023 [25]	32 F	Abdominal pain	Yes	Yes	—	Infarcted	Emergency	Open splenectomy	Recovered	
2023	Digumarthi et al., 2023 [26]	61 F	Chronic left lower abdominal pain with nausea and vomiting	Yes	Yes	1080° torsion	Infarcted	Elective (post‐stabilization)	Open splenectomy	Full recovery	Thrombocytosis, splenic + portal vein thrombosis
2024	Cui et al. 2024 [27]	16 F	Acute pain, infarction	Yes	Yes	720° counterclockwise	Infarcted	Emergency	Splenectomy	Recovered	
2025	Wubie et al. 2025 [28]	40 F	Acute abdomen	Yes	Yes	1080° counterclockwise torsion of splenic pedicle	infarcted	Emergency	Open splenectomy	Recovered	Momentum + small bowel adhesions
2025	Kashbour et al., 2025 [29]	34 F	Acute left iliac fossa pain, vomiting	Yes	Yes	—	Infarcted	Emergency	Open splenectomy	Full recovery	—

Imaging plays a central role in diagnosing wandering spleen (WS) with abdominal ultrasound often being the initial modality as shown by Karmazyn et al. [31], while cross‐sectional imaging, particularly contrast‐enhanced CT angiography, is critical for identifying splenomegaly, ectopic splenic positioning, and torsion of the vascular pedicle. In our case, prior CT imaging revealed a pelvic spleen with intact vasculature and an epithelial cyst, findings that have also been reported in previous studies [31]. The classic “whirl sign” confirmed torsion with maintained perfusion supporting elective surgery rather than emergency splenectomy as required in cases by Wubie et al. and Thorpe et al. [13, 28].

Pancreatic tail involvement in splenic torsion is anatomically feasible due to its close connection via the splenorenal ligament. Prior reports, such as those by Wang et al. and Colombo et al., confirmed that splenic torsion can lead to pancreatic traction, obstruction, or inflammation [7, 32]. Our case aligns with this mechanism, presenting intermittent, positionally triggered pancreatitis with radiologic evidence of torsion and elevated lipase, yet without infarction allowing for elective robotic splenectomy. While infarcted or ischemic spleens require emergent splenectomy, patients with preserved perfusion and stable vitals as in our case can benefit from elective intervention, through either splenectomy or splenopexy [33]. Comparative literature supports elective splenopexy or splenectomy in stable patients [7, 11, 14, 15], but few reports document pancreatic involvement, and none describe our case presentation with radiologic torsion and pancreatic ischemia, underscoring the clinical relevance of documenting this rare presentation.

## 6 Conclusion

Patients with wandering spleen can have chronic recurrent abdominal pain, ischemia, or secondary pancreatitis due to torsion of the splenic pedicle and compression of the pancreatic tail. CT angiography is essential for diagnosis. Management requires multidisciplinary input; elective surgery is appropriate in stable patients, while splenectomy is indicated in cases of infarction. Clinicians should consider wandering spleen in patients with recurrent pancreatitis and abnormal splenic position on imaging. Significant organ mobility can create intraoperative challenges, highlighting the need for meticulous surgical planning, particularly when using robotic approaches.

## Author Contributions

**Mohamed Nasser Elshabrawi:** writing – original draft. **Ismail Elkhattib:** conceptualization, data curation, supervision, writing – review and editing. **Dina Elraggal:** writing – original draft. **Bassant Elsayed:** visualization, writing – review and editing. **Neil Hansen:** data curation, supervision, writing – review and editing. **Faruq Pradhan:** supervision, writing – review and editing.

## Funding

The authors have nothing to report.

## Consent

Written informed consent was obtained from the patient for publication of this case report and accompanying images.

## Data Availability

The datasets generated and analyzed during the current study are not publicly available due to patient privacy and confidentiality concerns as per institutional ethics guidelines. However, deidentified data may be made available from the corresponding author upon reasonable request with appropriate ethical approval and institutional authorization.
